# New Horizons in IgA Nephropathy: Approach to Current and Emerging Therapies

**DOI:** 10.7759/cureus.107347

**Published:** 2026-04-19

**Authors:** Sai Kumar Madhavaram, Samyuktha Srinivas, Sarah A Bhanushali, Kartik Kalra

**Affiliations:** 1 Medicine, Manipal Academy of Higher Education, Manipal, IND; 2 General Medicine, Manipal Academy of Higher Education, Mangalore, IND; 3 Psychology and Neuroscience, Temple University, Philadelphia, USA; 4 Nephrology, Geisinger Medical Center, Danville, USA

**Keywords:** b-cell targeted therapy, complement inhibition, emerging therapies, endothelin receptor antagonists, iga nephropathy, targeted-release budesonide

## Abstract

IgA nephropathy (IgAN) is the most common primary glomerulonephritis globally, exhibiting marked diversity in clinical presentation, risk of progression, and response to therapy. Despite advances in understanding its pathophysiology, including the pivotal role of galactose-deficient IgA1 (Gd-IgA1), anti-Gd-IgA1 autoantibodies, and dysregulated mucosal and systemic immune responses, therapeutic options remain limited. This review provides a comprehensive overview of the current therapeutic landscape in IgAN, which is stratified into conservative care with renin-angiotensin-aldosterone system (RAAS) inhibitors, sodium-glucose cotransporter-2 (SGLT2) inhibitors, and corticosteroids, and newer advances like the endothelin receptor antagonists, immunosuppressive agents, and targeted therapeutics. Through synthesizing evidence from recent clinical trials and ongoing studies, we aim to provide clinicians and researchers with a current and forward-looking perspective on therapeutic strategies in IgAN, emphasizing the shift from conservative therapy to targeted, individualized interventions.

## Introduction and background

IgA nephropathy (IgAN) stands as the most prevalent primary glomerulonephritis worldwide, characterized by considerable heterogeneity in presentation, progression, and response to therapy. The disease is characterized by mesangial deposition of IgA-containing immune complexes and displays striking heterogeneity in its clinical presentation, ranging from isolated microscopic hematuria to rapidly progressive glomerulonephritis. Over the past decade, a comprehensive understanding of its pathogenesis has caused a paradigm shift from non-specific supportive care to targeted precision therapies. This comprehensive review outlines the evolution of IgAN management, highlighting landmark clinical trials and newer therapeutic agents.

Full remission of IgAN includes three aspects. Alongside complete remission of proteinuria, stabilization of estimated glomerular filtration rate (eGFR) with its annual loss corresponding to the physiological loss of approximately 1 ml/min per year in older adults or nil in younger adults is required. The absence of persistent microhematuria is the third criterion for fulfilling the definition of complete remission. There is emerging evidence that the extent and persistence of hematuria are important indicators in the progression of IgAN [1]. Recent clinical trials have demonstrated that the disappearance of microhematuria with complete remission of proteinuria and stabilization of eGFR has been seen in a significant proportion of patients and can become a realistic goal in the treatment of IgAN [2-4]. It is worth noting that an absence of hematuria does not mean that there is no ongoing inflammation and organ destruction. Hence, patients without hematuria must not be denied treatment with drugs designed to reduce the pathological inflammation through IgA immune complex deposition. The NefIgArd trial showed benefit from targeting pathogenic IgA production irrespective of whether they had hematuria [2]. In comparison, persistent microhematuria in the absence of significant proteinuria may not be associated with a poor prognosis and calls for verifying technical issues that may be related to the quantification of hematuria.

Mitigation of nephron loss with renoprotective therapies such as renin-angiotensin-aldosterone system (RAAS) blockade, and adjuncts like sodium-glucose cotransporter-2 (SGLT2) inhibitors and endothelin-1 receptor antagonists optimizes blood pressure (goal ≤120/70 mmHg) and helps reduce proteinuria and preserve glomerular filtration rate (GFR). Control of glomerular inflammation in patients with active inflammatory lesions and significant proteinuria is achieved through immunosuppressive therapy in the form of systemic corticosteroids and non-steroidal immunosuppressants. This can dampen glomerular inflammation and curtail acute injury. Novel and targeted therapies like complement inhibitors may offer an alternative anti-inflammatory approach by blocking the IgA-induced complement activation that drives glomerular damage. Reduction of pathogenic IgA production with therapies aimed at B cells and plasma cells can reduce the source of immune complexes, essentially the center point and crux of the pathogenesis of IgAN. Novel targeted therapies like targeted-release budesonide delivered to gut lymphoid tissue can decrease galactose-deficient IgA1 (Gd-IgA1) production with minimal systemic exposure. Since there are no anti-fibrotic therapies yet available, specifically for IgAN, future treatments may aim to block pro-fibrotic pathways in the kidney. A combination of therapies targeting different mechanisms may be needed to achieve optimal disease control. Further on, we discuss each modality in detail.

**Figure 1:**
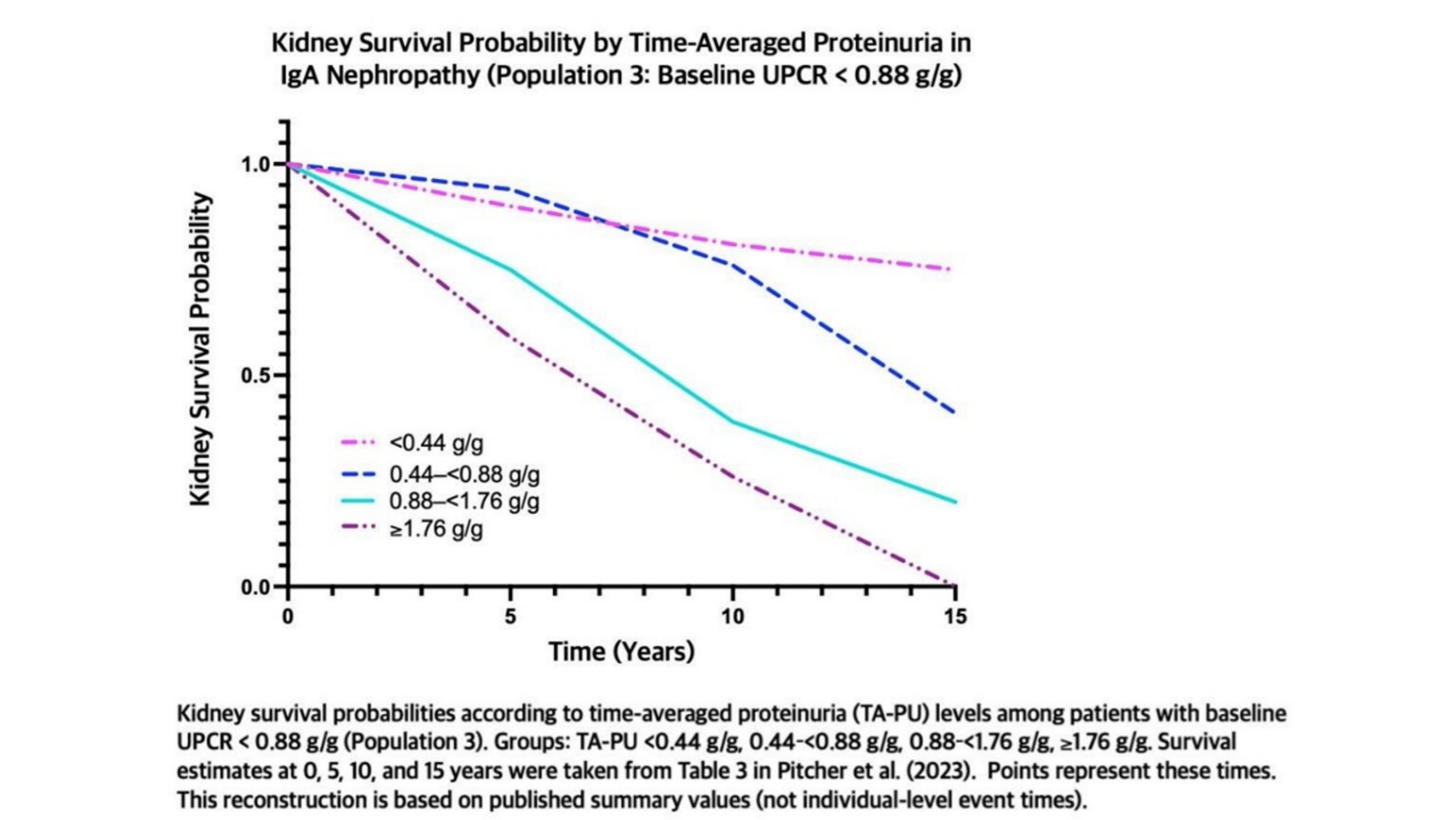
Kidney survival probability by time-averaged proteinuria in IgA nephropathy. Figure created by the authors. Data adapted from Pitcher et al. [5] and the KDIGO IgA Nephropathy Work Group: KDIGO 2025 Clinical Practice Guideline for the Management of Immunoglobulin A Nephropathy (IgAN) and Immunoglobulin A Vasculitis (IgAV) [6] under the terms of the Creative Commons Attribution-NonCommercial-No Derivatives License (CC BY NC ND). UPCR: urine protein-to-creatinine ratio.

Figure 1 shows kidney survival probability over 15 years in patients with IgAN and low baseline proteinuria (urine protein-to-creatinine ratio (UPCR) < 0.88 g/g), grouped by time-averaged proteinuria levels. Higher sustained proteinuria is associated with a more rapid decline in kidney survival, while lower proteinuria preserves kidney function for longer periods. Four groups are compared (<0.44, 0.44-<0.88, 0.88-<1.76, and ≥1.76 g/g), illustrating that prognosis worsens as proteinuria increases.

## Review

Treatment approaches: Multi-pronged treatment approach for IgA nephropathy

**Figure 2:**
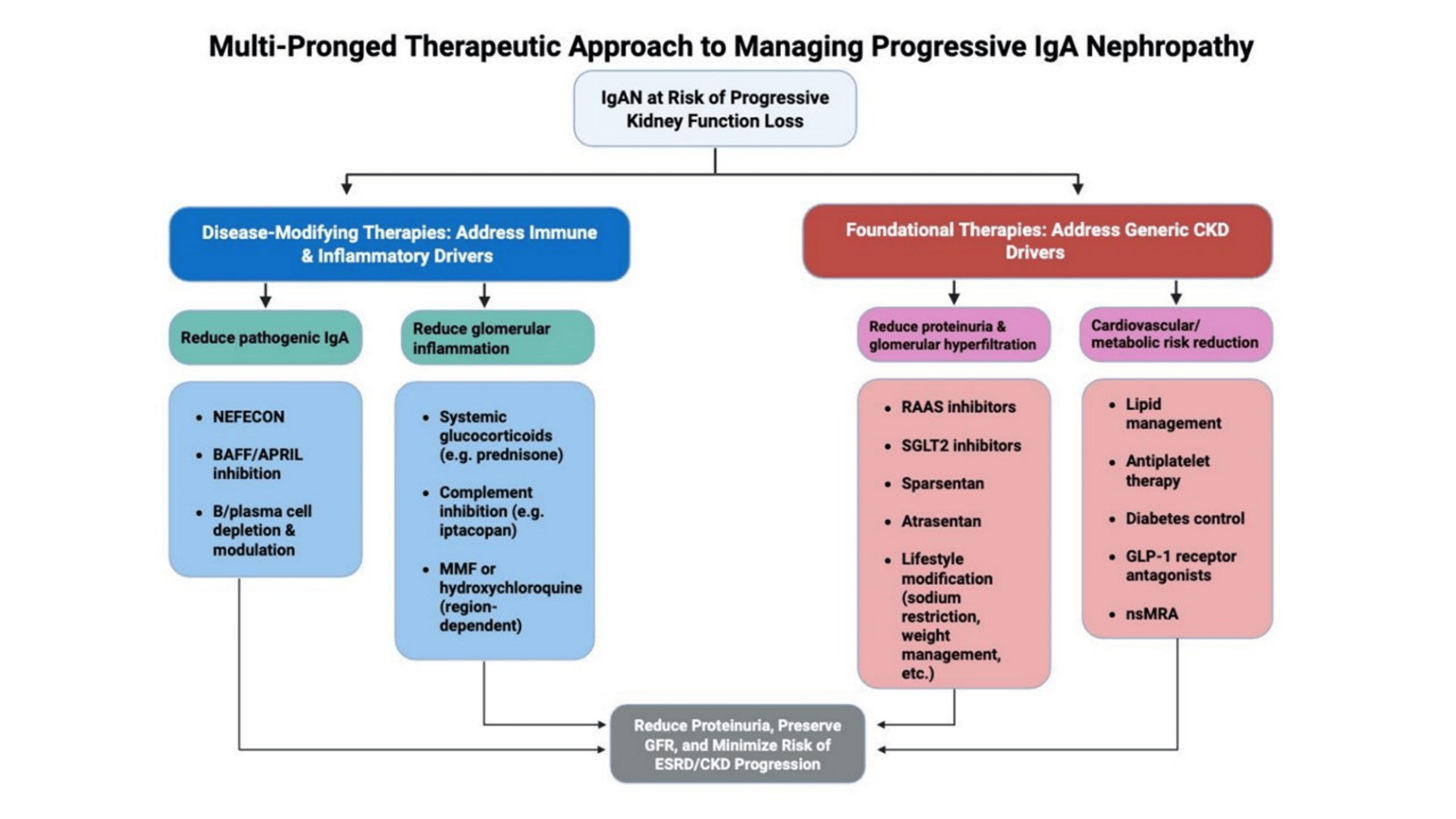
Multi-pronged therapeutic approach to managing progressive IgA nephropathy. Figure created by the authors. Adapted from the KDIGO IgA Nephropathy Work Group: KDIGO 2025 Clinical Practice Guideline for the Management of Immunoglobulin A Nephropathy (IgAN) and Immunoglobulin A Vasculitis (IgAV) [6] under the terms of the Creative Commons Attribution-NonCommercial-No Derivatives License (CC BY NC ND). IgAN: immunoglobulin A nephropathy; NEFECON: targeted-release budesonide; BAFF: B-cell activating factor; APRIL: a proliferation-inducing ligand; CKD: chronic kidney disease; RAAS: renin-angiotensin-aldosterone system; SGLT2: sodium-glucose cotransporter-2; GLP-1: glucagon-like peptide-1; MMF: mycophenolate mofetil; GFR: glomerular filtration rate; ESRD: end-stage renal disease; nsMRA: non-steroidal mineralocorticoid receptor antagonist.

**Figure 3:**
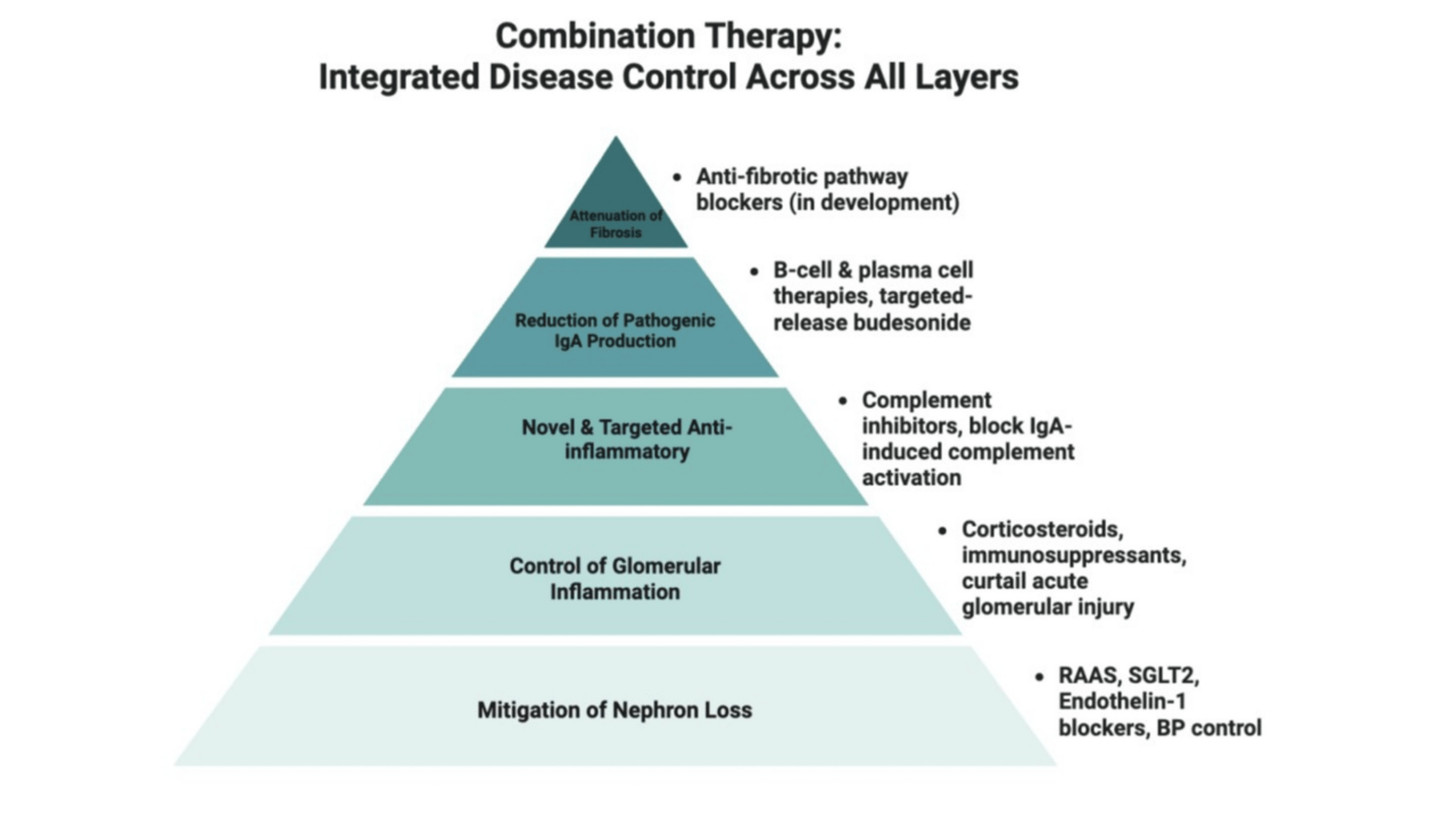
Combination therapy: Integrated disease control across all layers. Figure created by the authors. Adapted from the KDIGO IgA Nephropathy Work Group: KDIGO 2025 Clinical Practice Guideline for the Management of Immunoglobulin A Nephropathy (IgAN) and Immunoglobulin A Vasculitis (IgAV) [6] under the terms of the Creative Commons Attribution-NonCommercial-No Derivatives License (CC BY NC ND). RAAS: renin-angiotensin-aldosterone system; SGLT2: sodium-glucose cotransporter-2; BP: blood pressure.

Management of IgAN has evolved from a universally generalized supportive approach to a personalized strategy that combines supportive care with the use of immunosuppressives and disease-modifying therapies in selected patients. The goals of therapy are to reduce ongoing kidney inflammation and injury, achieve remission of proteinuria and hematuria, stabilize eGFR, and ultimately prevent progression to end-stage renal disease (ESRD). Given the complex pathogenesis of IgAN, a multi-model approach is needed to address all aspects of the disease. For better understanding and optimization, treatment of IgAN can be divided into foundational or reno-protective therapies, which prevent progression of chronic kidney disease (CKD), and disease-modifying or targeted therapies that attenuate disease activity and treat the immunological disease. The management of IgAN rests on a foundation of optimal supportive care, upon which additional therapies that target specific pathogenic pathways are added. Figures 2, 3 depict the multi-pronged treatment approach to managing IgAN.

Therapeutic Targets and Multimodal Management Strategies in IgA Nephropathy

Mitigation of nephron loss with renoprotective therapies such as RAAS blockade, and adjuncts like SGLT2 inhibitors and endothelin-1 receptor antagonists, which optimize blood pressure (goal ≤120/70 mm Hg) and help reduce proteinuria and preserve GFR.

Control of glomerular inflammation in patients with active inflammatory lesions and significant proteinuria with immunosuppressive therapy in the form of systemic corticosteroids and non-steroidal immunosuppressants. This can dampen glomerular inflammation and curtail acute injury. Novel and targeted therapies like complement inhibitors may offer an alternative anti-inflammatory approach by blocking the IgA-induced complement activation that drives glomerular damage.

Reduction of pathogenic IgA production with therapies aimed at B cells and plasma cells can reduce the source of immune complexes, essentially the center point and crux of the pathogenesis of IgAN. Novel targeted therapies like targeted-release budesonide delivered to gut lymphoid tissue can decrease Gd-IgA1 production with minimal systemic exposure.

Attenuation of fibrosis: Since there are no anti-fibrotic therapies yet available specifically for IgAN, future treatments may aim to block pro-fibrotic pathways in the kidney. A combination of therapies targeting different mechanisms may be needed to achieve optimal disease control. Further on, we discuss each modality in detail.

Treating Chronic Kidney Disease

Patients with immune-mediated IgAN often develop CKD [7]. This supportive kidney therapy in CKD, which is not specific to IgAN, constitutes a key component of the care. They encompass measures ranging from lifestyle modification, smoking cessation, and blood pressure control to pharmacologic therapy. They work on managing the intrarenal response to IgAN-induced complications such as glomerular hypertension/hyperfiltration, the tubulointerstitial response to persistent proteinuria, and the initiation or worsening of systemic hypertension [7].

Lifestyle and dietary modifications: Conservative therapy forms the base for every IgAN patient, regardless of disease severity. A low-sodium diet (<2 g/day of sodium) is generally adopted for blood pressure control and to reduce fluid retention [8]. Maintaining a healthy weight with regular exercise and smoking cessation is recommended, as obesity and smoking can worsen proteinuria and progression of kidney injury through hemodynamic stress and microvascular injury. Nephrotoxic medications like nonsteroidal anti-inflammatory drugs are generally avoided [9]. Some evidence suggests moderate protein restriction up to 0.8 g/kg/day dietary protein and vigorous glycemic control with coexistent diabetes could slow down the progression of CKD [10].

Blood pressure control: Strict blood pressure control is paramount in IgAN as a measure to reduce proteinuria and progression to CKD. The Kidney Disease: Improving Global Outcomes (KDIGO) 2021 guidelines recommend a target blood pressure of <120/80 mmHg for proteinuric CKD patients. The same has been associated with better outcomes in IgAN [8]. RAAS blockade is the first-line antihypertensive strategy. Additional agents like diuretics, dihydropyridine calcium channel blockers, beta-blockers, and new agents like endothelin receptor antagonists are added as needed to reach the goal blood pressure. The agents used to control blood pressure are discussed below.

RAAS inhibition: Angiotensin-converting enzyme inhibitors (ACEIs) or angiotensin receptor blockers (ARBs) constitute the first-line antihypertensives in all patients with IgAN, regardless of the presence of hypertension, and are recommended at any level of proteinuria [11]. Multiple studies have suggested that renin-angiotensin system (RAS) blockade improves outcomes in patients with IgAN. Its associations with reduction in proteinuria and inflammatory markers, along with its antifibrotic effects, have a favorable effect on kidney profile, with improved survival in proteinuric kidney disease [12,13]. Its benefit among normotensive individuals with only moderately increased proteinuria (around 0.5 g/day) remains unclear.

Smaller clinical trials about 20 years ago had demonstrated additional antiproteinuric effects through the co-administration of ACEI and ARB in IgAN patients [14]. However, recent findings from the STOP-IgAN cohort disagree with the dual RAS blocker regimen. Patients on dual RAS blockade had high proteinuria at the end of the three-year randomized controlled trial (RCT), whereas overall renal outcomes were comparable between participants under single and dual RAS blocker therapy [15]. A 2011 Cochrane review on non-immunosuppressive therapies, including antihypertensives, fish oil, antiplatelet/anticoagulants, and tonsillectomy for IgAN, concluded that antihypertensive therapy, primarily with RAS blockade, was the only non-immunosuppressive therapy to demonstrate clinical benefit in IgAN [7].

Another RAS-blocking therapy with the direct renin inhibitor, aliskiren, when combined with ARB treatment, has been shown to reduce proteinuria by a further 26% at six months and suppress serum interleukin-6 (IL-6) and transforming growth factor-beta (TGF-β) levels in IgAN patients with proteinuria > 1 g/day [16]. However, in current clinical practice, aliskiren has not been commonly utilized due to a high incidence of hyperkalemia and hypotension among type 2 diabetics with CKD [9]. Current guidelines recommend that patients with IgAN with proteinuria >0.5 g/day be placed on maximally tolerated RAS blockade with an ACE inhibitor or ARB, even when normotensive [8].

SGLT2 inhibitors: SGLT2 inhibitors have emerged as a valuable adjunct in CKD and IgAN. These drugs reduce proximal tubular sodium reabsorption, thereby lowering glomerular hyperfiltration, while also exhibiting anti-inflammatory and diuretic effects. In the Dapagliflozin and Prevention of Adverse Outcomes in Chronic Kidney Disease (DAPA-CKD) trial, which enrolled CKD patients of various etiologies, a subgroup of 270 IgAN patients with a low median eGFR of 40 ml/min showed significant benefit from dapagliflozin combined with RAS [17]. Renal outcome markedly improved, and the hazard ratio for the renal endpoint (50% loss of eGFR, dialysis, or death from a kidney disease-related or cardiovascular cause) was 0.29 (95% CI: 0.12, 0.73) compared to placebo [18]. Only 4% of IgAN patients on dapagliflozin reached the endpoint vs. 15% on placebo. The Canagliflozin and Renal Outcomes in Type 2 Diabetes and Nephropathy (CREDENCE) trial demonstrated that the addition of canagliflozin to standard of care improved kidney and cardiovascular outcomes in patients with type 2 diabetes and kidney disease [19]. The Empagliflozin in Patients with Chronic Kidney Disease (EMPA-KIDNEY) trial demonstrated that the addition of empagliflozin slows CKD progression and reduces cardiovascular mortality in both diabetic and nondiabetic kidney disease [20]. Together, these studies demonstrate the benefit of SGLT-2 inhibitors on both diabetic and nondiabetic CKD as part of the supportive therapy regimen in patients with IgAN, proteinuria, and CKD.

It has not been demonstrated yet whether SGLT-2 inhibitor therapy will benefit patients with preserved eGFR, as these subsets of patients were not included in the DAPA-CKD or EMPA-KIDNEY trials [21]. The ideal timing of SGLT-2 inhibitor initiation is also uncertain in the setting of IgAN, as it appeared most effective as a late intervention in these trials. The mean age of the patients in the DAPA-CKD trial was 61.8 years, and in the EMPA-KIDNEY trial was 63.8 years. This, along with reduced eGFR in enrolled patients, suggests the patients may have had significant disease chronicity at the time of entry into the study [21].

The concomitant use of SGLT-2 inhibitors with corticosteroids is also under scrutiny, due to the increased risk of urogenital infections seen in diabetic patients on SGLT-2 inhibitors. Although the DAPA-CKD or EMPA-KIDNEY trials did not show an increased risk of urogenital infections, patients receiving immunosuppressive therapy were excluded in both [20,22]. One approach that may be used in clinical practice may be to add an SGLT-2 inhibitor after completion of immunosuppression [23].

Treating Immunologic Disease With Immunosuppressive Therapy

In patients with IgAN, there is a growing emphasis on not waiting for evidence of progression; instead, initiating upfront therapy with both foundational measures and disease-modifying strategies is being advocated. The goal of immunosuppression is to dampen the glomerular immune response, reduce ongoing inflammation, and preserve renal function. Because IgAN often has a prolonged course, the decision to use immunosuppressives must balance potential benefits with substantial risks.

A standard pathogenic scheme is followed in most immune-mediated glomerular diseases. Deposition of immune complexes in the renal mesangium leads to complement activation and cellular activation. This attracts leukocytes and pro-inflammatory mediators, which produce profibrotic signals. The spreading of the primary glomerular injury to the tubulointerstitium with subsequent tubular damage and interstitial fibrosis leads to irreversible nephron loss. Keeping this pathogenesis in mind, three goals of immunologic management of IgAN can be established: (i) stop the production of pathogenic forms of IgA and IgA immune complexes; (ii) halt the glomerular inflammation to prevent or attenuate irreversible parenchymal damage; (iii) halt the release of profibrotic signals in glomeruli and the tubulointerstitium to prevent irreversible nephron loss.

Corticosteroids: Systemic glucocorticoids have been the mainstay of immunosuppressive treatment in IgAN since it was first described as an autoimmune, immune complex-mediated glomerulonephritis. High-dose prednisone can induce remission of proteinuria in many patients, presumably by suppressing immune complex-mediated inflammation. Early studies suggested that steroids slow down the loss of kidney function in IgAN. Although corticosteroids have been the most widely studied agents, evidence supporting their use remains inconclusive, with debates around both their efficacy and safety [24]. There is significant variability across trials in interpreting the benefits of steroid therapy in IgAN, dosing schedules, patient demographics, and concomitant use of supportive treatments, such as RAS inhibitors. Extended use of steroids for >1 year has not been shown to be more beneficial in comparison to moderate-term regimens over six to nine months [25].

Earlier clinical practice validated the use of steroids up to 1 mg/kg/day tapered over six months or pulse dosing every other month [26]. However, the STOP-IgAN RCT challenged this perspective. In patients with IgAN and substantial proteinuria, the STOP-IgAN trial found no long-term benefit of adding immunosuppressive agents (steroids plus non-steroidal immunosuppressants) in addition to conservative measures on hard outcomes (serum creatinine, proteinuria, end-stage kidney disease (ESKD), and death), over a 10-year follow-up [27]. The TESTING trial saw that high-dose oral methylprednisolone (0.8-1 mg/kg/day) significantly reduced the risk of the composite outcome (eGFR decline, ESRD, or death) by about 50% compared to placebo. But it came at an unacceptable cost. A total of 14.7% of patients on methylprednisolone experienced major adverse events, compared to just 3.2% in the placebo group, with 8.1% suffering severe infections (including two deaths). This led to the trial’s suspension. A follow-up TESTING Low-Dose study was introduced with a reduced steroid regimen (0.4 mg/kg/day for two months, tapered over six to nine months with prophylactic antibiotics). While adverse events in the low-dose group were nearly half of those in the high-dose group, the steroid group still reported about four times more serious events than the placebo [28]. Although corticosteroids provided benefit, the incidence of severe and even fatal infections outweighed the modest gains in proteinuria reduction and renal preservation [28].

Currently, targeted-release formulation (TRF) budesonide is the only immunosuppressive drug approved by the FDA to treat IgAN. TRF budesonide is said to be delivered directly to the Peyer patches, where the galactose-deficient IgA is produced, thus interrupting a key mediator of IgAN. The NefIgArd (Efficacy and Safety of Nefecon in Patients With Primary IgA Nephropathy) trial evaluated the efficacy of TRF in patients with proteinuria of 1 g or more over a nine-month period. TRF budesonide resulted in significantly reduced proteinuria, and the eGFR benefit was sustained over a two-year follow-up. However, the proteinuria recurred after TRF budesonide was stopped, and steroid-related side effects were more common in the TRF budesonide group [2]. In summary, corticosteroids may provide short-term benefit in selected patients, but their use comes with significant risk of toxicity. Careful patient selection, screening for infections, and pre-treatment vaccinations are essential.

Non-steroid immunosuppressants: Non-steroidal immunosuppressants play a selective and adjunctive role in the management of IgAN rather than serving as first-line therapy. Their use is typically guided by disease severity, risk of progression, and patient-specific factors, with certain agents reserved for aggressive or rapidly progressive disease and others considered in cases of persistent proteinuria despite optimized supportive care. Importantly, the evidence supporting these therapies varies across populations, highlighting the need for individualized, context-driven treatment decisions.

Novel and Targeted Therapies in IgA Nephropathy

Table 1 outlines the evidence and recommendations for non-steroid immunosuppressant drugs in IgAN management. It categorizes four drug classes (mycophenolate mofetil (MMF), cyclophosphamide + azathioprine, calcineurin inhibitors, and hydroxychloroquine) and provides their evidence base as well as clinical recommendations. The table highlights that MMF shows positive results in Chinese RCTs but is not recommended for Western patients. Cyclophosphamide with azathioprine is reserved for rapidly progressive IgAN or systemic vasculitis. Calcineurin inhibitors are not routinely recommended due to nephrotoxicity and uncertain long-term benefits. Hydroxychloroquine may reduce proteinuria and can be considered for persistent cases in Chinese patients. Each drug’s recommendation is tailored to the evidence and population context described.

**Table 1:** Non-steroid immunosuppressants in IgA nephropathy. Created by the authors. RCT: randomized controlled trial; IgAN: IgA nephropathy; RAS: renin-angiotensin system.

Drug/class	Evidence summary	Clinical recommendation
Mycophenolate mofetil (MMF)	• Positive results in 3 Chinese RCTs: ↓ proteinuria, preserved kidney function • Negative in 3 Western RCTs: No benefit or worse outcomes	May be used as a steroid-sparing agent in Chinese patients. Not generally recommended in Western populations [28].
Cyclophosphamide + azathioprine	• UK RCT: Improved kidney survival in crescentic IgAN • STOP-IgAN trial: ↑ adverse events, no long-term benefit	Reserved for rapidly progressive IgAN with crescents or systemic vasculitis. Consider in children with severe disease [29].
Calcineurin inhibitors (tacrolimus, cyclosporine)	• Short-term proteinuria reduction • High relapse on discontinuation; nephrotoxic • Meta-analysis: Tacrolimus + steroids ↓ proteinuria	Not routinely recommended due to uncertain long-term benefit and toxicity [30].
Hydroxychloroquine (HCQ)	• Chinese RCT: ↓48% proteinuria at 6 months vs. 10% in placebo • No major adverse events reported	Can be considered in Chinese patients with persistent proteinuria despite RAS blockade [31,32].

The treatment of IgAN has undergone a decade-long transformation based on an evolving appreciation of pathogenesis and regulatory endorsement of surrogate markers such as proteinuria and slope of eGFR. Several newer agents that target distinct immunologic, inflammatory, and complement-mediated mechanisms have entered the arsenal. These are a long way from traditional corticosteroids and RAAS blockade and represent a new era of precision nephrology [33].

Targeted-release budesonide (Nefecon): Nefecon (Tarpeyo) is an oral targeted-release budesonide for delivering the corticosteroid directly to the distal ileum - a region with high densities of Peyer's patches and gut-associated lymphoid tissue (GALT), where the aberrant mucosal immune activation of IgAN occurs [34-37]. Budesonide is metabolized extensively in the first pass through the liver, limiting systemic exposure to corticosteroids but permitting local immunosuppression. The NefIgArd trial was a double-blind, placebo-controlled, multicenter, randomized, phase 3 trial enrolling IgAN patients with biopsy-proven IgAN, proteinuria ≥1 g/day, and eGFR of 35-90 mL/min/1.73 m² on stable RAAS blockade. Patients were treated with TRF-budesonide 16 mg/day for nine months. At nine months, the budesonide group had 27% decrease in proteinuria versus 2% on placebo. The mean eGFR decline was 3.8 mL/min/1.73 m² less than placebo at two-year follow-up, establishing a disease-modifying effect [36]. The approved clinical regimen is 16 mg oral taken as four 4 mg delayed-release capsules once daily for nine months. Patients experienced a rebound in proteinuria after cessation of therapy, indicating that mucosal suppression may need to be continued. About one-third of patients on TRF-budesonide developed corticosteroid-related side effects, including weight gain, acne, hypertension, and edema. These side effects were, however, less severe than those of systemic glucocorticoids [36,37]. On the basis of the positive phase 3 results, the US FDA approved in December 2021 on accelerated grounds and in 2023 on full grounds. Nefecon (under the brand name Tarpeyo) is now listed in guidelines as a targeted drug for treating patients with persistent proteinuria despite optimal supportive care [38]. At present, no head-to-head randomized trials have directly compared Nefecon with systemic corticosteroids, and this is a critical gap in evidence [36]. Pharmacokinetics have shown that 16 mg of Nefecon is approximately equivalent to 8 mg of prednisone in terms of plasma cortisol suppression, indicating a lower systemic steroid exposure compared to traditional systemic corticosteroids. Important to note that Nefecon cannot be substituted with other budesonide formulations, as its therapeutic effect relies on its unique beaded, targeted-release properties.

B-cell and plasma cell-directed therapies: The pathogenesis of IgAN is also directly attributed to dysregulated B-cell activation and production of Gd-IgA1 and anti-Gd-IgA1 autoantibodies. Therefore, therapies targeting B cells and plasma cells constitute a key strategy in the interference of the autoimmunity of IgAN. Two critical cytokines, BAFF (B-cell activating factor) and APRIL (a proliferation-inducing ligand), regulate B-cell biology at different stages. BAFF primarily promotes early B-cell survival, proliferation, differentiation, and immunoglobulin class switching, whereas APRIL influences later stages of B-cell maturation, including plasma cell survival, IgA production, and modulation of the gut mucosal immune axis. Interfering with these pathways may reduce pathogenic IgA1 production.

Sibeprenlimab is a monoclonal antibody that selectively blocks APRIL, a cytokine implicated in the pathogenesis of IgAN. By inhibiting APRIL, it aims to reduce plasma cell survival and the production of Gd-IgA1, thereby decreasing proteinuria and slowing down kidney function. The phase 2 ENVISION trial demonstrated a significant reduction in proteinuria compared to placebo, supporting its potential efficacy in IgAN treatment [9]. The phase 3 VISIONARY trial, which enrolled 530 patients, showed that treatment with sibeprenlimab led to a 51.2% reduction in proteinuria at nine months compared to placebo (P < 0.0001) [39]. In November 2025, sibeprenlimab received accelerated approval by the US FDA owing to its reduction of proteinuria in adults with primary IgAN at risk for disease progression.

Rituximab (anti-CD20 monoclonal antibody), a B-cell depleting drug to CD20, was investigated in IgAN based on its efficacy in other autoimmune glomerulopathies. However, a few trials, including a subset analysis of the TESTING study, showed no advantage in proteinuria reduction or kidney outcomes [38]. This may be attributed to the reduced ability of rituximab to deplete mucosal B cells or its failure to impact long-lived plasma cells, which lack the CD20 antigen [39].

Atacicept (dual BAFF/APRIL inhibitor) is a recombinant fusion protein that blocks BAFF and APRIL - two cytokines mediating B-cell survival, differentiation, and class switch to IgA. Weekly atacicept 150 mg in the ORIGIN phase 3 trial had a ~33% reduction in proteinuria compared to placebo at 24 weeks. It also decreased circulating Gd-IgA1 levels markedly. The drug also had an excellent safety profile, with phase 3 trials currently ongoing [40].

Telitacicept, another BAFF/APRIL dual inhibitor, has only been tested predominantly in the Chinese population. In a phase II trial, it led to proteinuria reductions and eGFR improvement (+2.3 mL/min/1.73 m² at six months), which made its continued development in IgAN appropriate. Phase III trials are ongoing in China and worldwide [41].

Felzartamab and mezagitamab (anti-CD38 monoclonal antibodies) bind to CD38, a marker found on long-lived IgA-secreting plasma cells. These treatments are hoped to decrease the chronic antigenic drive from uninhibited autoreactive plasma cells by rituximab. Felzartamab is in phase II trials, and previous trials with bortezomib (a plasma cell-depleting proteasome inhibitor) produced partial or complete remission in three of eight steroid-resistant IgAN patients [39,42].

Complement inhibitors: Atypical activation of the complement system, particularly the lectin and alternative pathways, is increasingly being recognized as a major mediator of glomerular injury in IgAN. Complement deposition (e.g., C3, C5b-9, and C4d) has been correlated with severity, proteinuria, and risk of progression. New treatments now target individual elements of these cascades to prevent inflammation mediated by the immune system.

Iptacopan (LNP023 - factor B inhibitor) is an oral factor B-selective inhibitor that plays a key role in the alternative complement pathway. By preventing amplification of C3 convertase, iptacopan inhibits downstream complement-mediated glomerular damage. In a phase 2 trial, iptacopan demonstrated a dose-dependent reduction in proteinuria in patients with chronic proteinuria. Interim analysis at nine months of the phase 3 trial (APPLAUSE-IgAN) replicated the effect and showed a trend toward improved eGFR preservation. It achieved a 44% reduction in proteinuria from baseline, compared to a 9% reduction in the placebo group, resulting in a 38% relative risk reduction (p < 0.0001). Fabhalta (iptacopan) was granted accelerated approval by the FDA in August 2024 for the reduction of proteinuria in adults with primary IgAN at risk of rapid disease progression [42].

Narsoplimab (anti-MASP-2 monoclonal antibody) blocks MASP-2, a serine protease that initiates the lectin complement pathway through C4 and subsequent effector activation. A phase II trial had provided evidence of a reduction in proteinuria and stabilization of eGFR. The ARTEMIS-IgAN phase III trial interim analysis in 2023, however, failed to hit pre-specified efficacy values, raising doubts about its future prospects [43]. However, MASP-2 is a desirable target, especially in patients with increased glomerular C4d or mannan-binding lectin (MBL) deposition.

Ravulizumab (C5 inhibitor), a long-acting anti-C5 monoclonal antibody, and cemdisiran, a subcutaneous small interfering RNA (siRNA) to inhibit hepatic C5 synthesis, are both terminal complement pathway inhibitors. Proteinuria is reduced, and acceptable safety has been shown in early-phase data, and both are being studied in the phase III SANCTUARY trial (NCT06291376) [37]. C5 blockade has widespread immunosuppressive potential but is at higher risk of infections, especially meningococcal disease, and needs vaccination and close monitoring.

Pegcetacoplan (C3 inhibitor) suppresses central C3 activation with pan-suppression of all three complement pathways. Pegcetacoplan is in an early-phase clinical trial in IgAN. Avacopan, an orally active C5a receptor antagonist (already licensed for antineutrophil cytoplasmic antibody (ANCA) vasculitis), should ideally minimize inflammation without terminal pathway blockade. The two drugs demonstrate theoretical advantage in IgAN, but need further proof [44,45].

Dual endothelin-angiotensin receptor blockade (DEARA), endothelin-1 (ET-1), is a powerful vasoconstrictor and pro-fibrotic peptide with roles in glomerular injury, inflammation, and proteinuria. Activation of the endothelin system is the cause of progressive renal damage in IgAN. Combined endothelin and angiotensin receptor antagonism provides a synergistic antiproteinuric and renoprotective effect. High-quality clinical trial data now exist to support this approach.

Sparsentan (dual endothelin type A (ETA)/ARB blockade) is an oral first-in-class molecule that blocks the ETA receptor and the angiotensin II type 1 receptor (AT1R) simultaneously. It was evaluated in the global phase 3 PROTECT trial in biopsy-proven IgAN patients with proteinuria >1 g/day and eGFR ≥30 mL/min/1.73 m² [8]. At 36 weeks, sparsentan caused a 49.8% reduction in proteinuria, versus 15.1% with irbesartan, the active control (p < 0.001). The overall eGFR slope difference at 110 weeks was not statistically significant (p = 0.058), but the chronic eGFR slope (excluding the acute hemodynamic dip in the first few weeks) was significantly better: -2.7 mL/min/1.73 m²/year with sparsentan vs. -3.8 mL/min/1.73 m²/year with irbesartan (p = 0.037) [46]. Sparsentan received full FDA approval in September 2024 for patients with IgAN and persistent proteinuria. It was approved under the brand name Filspari. This approval was based on the PROTECT study, a phase 3, head-to-head trial comparing sparsentan to irbesartan, which demonstrated that sparsentan significantly slowed kidney function decline over two years, as measured by eGFR, and provided durable reductions in proteinuria [8].

The FDA approved an important modification to the Risk Evaluation and Mitigation Strategy (REMS) for sparsentan in August 2025, simplifying safety monitoring requirements for patients with IgAN. Under the original REMS, patients were required to undergo monthly liver function test monitoring; however, following accumulated clinical trial and post-marketing safety data demonstrating a favorable hepatic safety profile, monitoring frequency was reduced to every three months during treatment. In addition, embryo-fetal toxicity-specific REMS requirements were removed, eliminating pregnancy-related REMS tracking and monitoring. Despite these changes, sparsentan continues to be dispensed through a restricted REMS program primarily due to the risk of hepatotoxicity, requiring prescribers, pharmacies, and patients to remain enrolled in the program.

Atrasentan is a selective endothelin A receptor antagonist that selectively inhibits the ETA-1 receptor. Being studied in the ALIGN trial, it is currently in phase 3 of clinical trial development for IgAN patients with residual proteinuria. Interim results of this study demonstrate a proteinuria decrease of about 38% at 12 weeks with good tolerability [36]. Based on these promising results, the FDA granted accelerated approval to Vanrafia (atrasentan) on April 2, 2025. This approval was for the reduction of proteinuria in adults with primary IgAN at risk of rapid disease progression, generally defined as a UPCR ≥1.5 g/g.

Gaps in knowledge

Despite recent achievements in the field of understanding and treatment of IgAN, several important knowledge gaps remain. The large-scale immunosuppression transition to pathophysiology-based targets has discovered new opportunities, but it has also uncovered areas of uncertainty that must be considered in future research [9]. The main difference is the lack of long-term data on new biological biology, sustainability, and comparative efficiency. Many new drugs, such as B-cell modulators, complement inhibitors, and target-release corticosteroids, have been prospectively tested in early-stage testing, but integration into long-term treatment models has not been validated [46]. Questions remain regarding optimal sequences, combined strategies, and duration of treatment. Another important gap is the need to identify reliable and well-validated biomarkers that can support individualized treatment planning [1].

While serum Gd-IgA1, anti-Gd-IgA1 autoantibodies, urinary MCP-1, CD89, and glomerular complement deposits (C3, C4d) show potential, none are currently validated for routine clinical use [3]. The predictive utility of these markers in response to treatment remains uncertain. Additionally, new tools are being studied, such as urinary exosome profiles and genetic markers (e.g., CFHR5 polymorphisms), but their clinical translation is still in its first stage [2]. There is also limited clarity about how treatments can be combined to obtain maximum control of the disease. Strategies followed by early immune regulation (e.g., BAFF/inhibition or BAFF/April inhibition), followed by complement inhibitors, are theoretically promising, but formal testing is required for controlled trials [40].

Furthermore, there is not sufficient actual data on efficiency, especially in different groups of populations and healthcare institutions. Most existing tests are performed in closely defined cohorts and have limited generalization [42]. Long-term observational studies are required to assess patient compliance, adverse event profiles, and kidney outcomes in patients other than replacement markers, such as proteinuria [46]. Finally, the economic efficiency and global availability of new treatment methods remain the main issues. IgAN is common in many countries with low incomes, but it can limit availability due to the high cost of biologics. Elimination of these knowledge gaps is important to optimize patient outcomes, guide clinical decisions, and create stable treatments for IgAN over the next decade [38].

Given the expanding therapeutic landscape in IgAN, a summary of FDA-approved therapies and investigational agents in advanced trials is presented in Table 2 and Table 3, respectively.

**Table 2:** FDA-approved therapies for IgA nephropathy. Created by the authors. ETA: endothelin type A receptor; AT1: angiotensin II type 1 receptor; ET-1: endothelin-1; eGFR: estimated glomerular filtration rate.

Therapy name	Target/mechanism	Key trial (Phase)	Main outcomes	FDA approval status
Nefecon (Budesonide)	Mucosal immune suppression via Peyer’s patches	NefIgArd (Phase 3)	↓27% proteinuria; stabilized eGFR	Full approval (December 2023) [6]
Sparsentan	Dual ETA and AT1 receptor blockade	PROTECT (Phase 3)	↓49.8% proteinuria; improved chronic eGFR slope	Full approval (September 2024) [7]
Iptacopan	Factor B inhibition (alternative complement pathway)	APPLAUSE (Phase 3)	~38% ↓ in proteinuria; eGFR stabilization	Accelerated approval (August 2024) [42]
Atrasentan	Selective ETA antagonist that inhibits the effects of ET-1.	ALIGN Trial (Phase 3)	Significant and clinically meaningful reduction in proteinuria vs. placebo	Accelerated approval (April, 2025) [27]

**Table 3:** Investigational therapies in advanced trials. Created by the authors. BAFF: B-cell activating factor; APRIL: a proliferation-inducing ligand; ETA: endothelin type A (receptor); RAAS: renin-angiotensin-aldosterone system; Gd-IgA1: galactose-deficient IgA1; eGFR: estimated glomerular filtration rate; UPCR: urine protein-to-creatinine ratio.

Treatment name	Class/target	Key clinical trials	Status	Notable findings/notes
Atacicept	BAFF + APRIL inhibition	ORIGIN-3 Trial	Phase 3 (ongoing)	↓33-35% reduction in proteinuria; significant reduction in Gd-IgA1 [39]
Telitacicept	BAFF + APRIL inhibition	TELIGAN Trial (NCT05799287)	Phase 3 (China/global)	Proteinuria reduction: −58.9% reduction in UPCR vs. −8.8% with placebo at ~39 weeks. Lower risk of ≥30% eGFR decline: 6.3% (telitacicept) vs. 27% (placebo) [40]
Sibeprenlimab	APRIL monoclonal antibody	VISIONARY Trial	Accelerated approval (2025)	Reduction in IgA and Gd-IgA1; 51.2% reduction in proteinuria at 9 months compared to placebo (P < 0.0001) [7,39]
Pegcetacoplan	Central C3/C3b inhibition	DISCOVERY Trial	Phase 2	~50.9% mean proteinuria reduction at 48 weeks in intent-to-treat (ITT) population and ~65.4% in the per-protocol (PP) population [44]
Avacopan	C5a receptor antagonist	CLASSIC-IgAN Trial	Phase 2	Reduction in complement-mediated inflammation: Targeted inhibition of C5a-driven inflammatory signaling [44]
Felzartamab	CD38 plasma cell depletion	IGNAZ Trial	Phase 2	Targets long-lived plasma cells; significant proteinuria reduction (~40–50% signal in interim analyses), and rapid decline in circulating immunoglobulins, including IgA [39]
Atrasentan	ETA receptor antagonists	ALIGN Trial	Accelerated approval (2025)	~38% reduction in proteinuria; alternative RAAS strategy [27]

Challenges and real-world management considerations

One Disease, Many Faces: The Challenge of Clinical Variability

IgAN presents in many guises, ranging from occult hematuria to rapid progressive glomerulonephritis. This variability renders choices of therapy challenging. Scoring systems like the Oxford classification and internet-based calculators provide a format, but cannot reliably predict with certainty which patients will stabilize and which will worsen. A synergy between personalized treatment, careful follow-up, and frequent reassessment deals with these uncertainties. This usually proves to be a challenge in real-time cases [38].

The Supportive Care Dilemma: When Is It Enough?

Guidelines recommend starting maximum supportive care therapy. In practice, though, it is faced with several challenges. Some of these factors cannot always be done in regular practice, which includes achieving blood pressure and proteinuria goals, which rely on patients' adherence, follow-up, and coordination [10]. Delays in immunosuppression in very aggressive cases and premature initiation in less risky patients remain a delicate balancing act [36].

Therapeutic Risks in Practice

The therapies for IgAN, such as corticosteroids and inhibitors of the complement system, are riddled with risks. Systemic steroids have well-documented side effects, particularly among young adults [6]. Even newer medications, such as budesonide, while safer overall, are not free of side effects [35]. Immunosuppressants and biologics carry risks for infection, decreased blood cell count, and long-term safety. Prevention measures, monitoring, and education of the patient need to be incorporated into every treatment plan in actual practice [30].

Treatment at a Cost

The potential of new therapies is overshadowed by their high cost. Medications like TRF-budesonide, sparsentan, iptacopan, and future biologics can be expensive, often limited by insurance or availability [38]. This disconnect risks creating a tiered system based on geography or socioeconomic status, where access to advanced treatment depends on one’s location or financial situation. Affordability and fairness will shape the future of IgAN treatment as much as effectiveness will [10].

Evidence in Progress

Most of the therapies used to treat IgAN are licensed based on short-term outcomes, primarily the degree to which they decrease urine protein. Although this is a helpful marker, long-term outcomes such as the extent to which the kidneys continue functioning with time or whether ESKD is avoided have not yet been widely researched [46]. Therefore, physicians must make treatment choices based on the best current evidence, early indication of benefit, and their clinical experience.

Global Differences in IgAN Treatment

Treatment of IgAN varies in most regions of the world. This is because every country has its own medical regulations, various drugs, and its own styles of curing disease [42].

For instance, in East Asia, physicians tend to administer steroid drugs early and in high concentrations. Some Japanese hospitals even remove tonsils during the course of treatment [10]. In China, MMF is routinely administered first, according to local tradition, although Western nations do not entirely agree with this [29].

Physicians in Western nations handle MMF more cautiously. They prescribe it primarily to East Asian patients or those who are unable to take steroids. Additionally, novel drugs such as SGLT2 inhibitors, endothelin antagonists, and complement inhibitors (such as iptacopan and narsoplimab) are not uniformly accessible, inducing unequal treatment access [19].

Due to these disparities, it is difficult to have a single world rule for the treatment of IgAN. That is why there is a need to involve individuals from various nations in their studies. Factors such as genes, environment, and healthcare systems all influence individuals in responding to treatment. New drugs need to be tried on various groups of individuals across the globe [46].

Sequencing and Combining Therapies

With many new drugs entering the arena, clinicians are now confronted with an incandescent question: Which therapy to employ, in what order, and in what combination? The guidelines today provide little direction [11]. Do endothelin antagonists precede or follow budesonide? Is the combination of complement inhibitors and B-cell therapies additive? Unless combination trials provide better direction, nephrologists will be forced into individualized, trial-and-error approaches in all too frequently uncertain circumstances [10].

The Limitations of Disease Monitoring

Unlike most glomerular diseases, IgAN lacks powerful non-invasive biomarkers to monitor disease activity or treatment response. Practitioners are thus forced to resort to surrogate markers like urine protein, eGFR change, and blood pressure, which do not reflect underlying tissue alterations. Biopsy is not always a routine matter, but fibrosis can creep forward. Experimental urinary markers like CD89, Gd-IgA1, or exosomal micro ribonucleic acids (miRNAs) are promising but not yet routine [3]. Until then, patient compliance with follow-ups and routine long-term monitoring is crucial [10].

Clinical Situations

The treatment of IgAN usually becomes challenging or unusual. Adult Henoch-Schönlein purpura due to IgA vasculitis in adults may include systemic features like purpura, arthritis, or gastrointestinal manifestations. This would then incline toward more aggressive immunosuppressive therapy in line with ANCA vasculitis treatment protocols [12]. Recurrent IgAN after transplant is a problem affecting almost one-third of kidney grafts and can cause dysfunction in the graft; its prevention is more than just the regulation of blood pressure and proteinuria. Crescentic IgAN could therefore require direct clinical intervention, irrespective of biopsy confirmation, if rapidly progressive glomerulonephritis is considered. Such a scenario highlights the need for clinical flexibility rather than stringent compliance with guidelines [42].

Management of IgAN Goes Beyond Biochemical Indices

Treatment side effects, long duration of disease, and uncertainty about prognosis can potentially damage patients' mental health and quality of life [25]. Physical changes such as steroid-induced moon facies or frequent infections may lead to stigma or depression. Decisions regarding the use of immunosuppression or costly therapy need to be based on shared decision-making, considering disease severity and patient preferences, lifestyle, and psychosocial situation. Patient education with information, e.g., risk calculators or symptom monitoring, can assist with participation and long-term adherence [10].

Future directions and research

New risk stratification approaches in IgAN aim to integrate molecular markers with clinical and histologic data, improving predictive precision and individualization of care. Serum levels of Gd-IgA1, IgA/C3 ratios, and polygenic risk scores are currently under investigation as part of this evolving framework [45]. In addition, the application of machine learning to large IgAN cohorts has shown potential to enhance predictive ability by identifying novel risk patterns beyond traditional clinical indicators. Noninvasive biomarkers, such as urinary chemokines, CD89, and exosomal microRNAs, are also being explored as “liquid biopsy” surrogates for disease activity and treatment monitoring [1].

Given that mucosal immune dysregulation is central to IgAN pathogenesis, new therapies are increasingly focused on modulating mucosal immune responses to restore tolerance and reduce aberrant IgA activation. Strategies such as oral mucosal tolerization, mucosal vaccination, and microbiome-directed therapy are being actively investigated [12]. Early trials involving probiotic supplementation have demonstrated modest immunomodulatory benefits, but these approaches remain largely experimental and require further validation before clinical translation [46].

Simultaneously, ongoing research continues to broaden the therapeutic landscape by identifying new druggable targets in IgAN. Among these are T-cell co-stimulatory molecules such as OX40/OX40L, signaling intermediates in the B-cell receptor pathway such as Bruton’s tyrosine kinase (BTK), and cytokines like IL-6, which play key roles in perpetuating glomerular inflammation [34]. Anti-fibrotic agents targeting TGF-β are also under active evaluation, though their use is tempered by concerns regarding systemic toxicity. In the future, molecular renal biopsy may enable personalized therapy selection, allowing clinicians to determine whether a patient is more likely to respond to complement blockade or immunosuppression [46].

Because IgAN is a multi-hit disease involving both immune and non-immune mechanisms, combination therapy represents a logical next step in disease management. Current clinical trials are exploring regimens that combine budesonide with SGLT2 inhibitors or corticosteroids with complement inhibitors to achieve synergistic reno-protection [8]. Further studies are anticipated to stratify patients based on biomarker-defined profiles, helping to determine not only which combinations are most effective but also the optimal sequencing of therapy [10].

Long-term modification of disease activity remains a major therapeutic goal, particularly as IgAN typically begins in young adulthood and may progress insidiously over decades. Novel treatments that induce sustained immune tolerance, such as peptide-based immunotherapies, represent an exciting frontier in this regard. Although these strategies are still experimental, they hold the potential to alter the natural course of the disease by addressing its immunologic foundations rather than merely suppressing downstream injury [46].

The refinement of clinical trial endpoints is another critical development in IgAN research. Because the disease often progresses slowly, traditional endpoints such as ESRD or major declines in eGFR require prohibitively long follow-up periods. While surrogate endpoints like proteinuria are commonly used, they remain imperfect predictors of outcome. Ongoing studies aim to validate intermediate markers such as iohexol-based GFR measurements, MRI-based fibrosis imaging, and eGFR slopes adjusted for proteinuria [7]. The advent of adaptive and platform trial designs may further accelerate therapeutic discovery by allowing simultaneous evaluation of multiple interventions within a single framework [10].

The overlap between IgA vasculitis (IgAV) and IgAN represents another area of active investigation. Both conditions share key pathogenic mechanisms, including the formation of Gd-IgA1 immune complexes, and consequently may respond to similar therapeutic approaches. Current clinical trials increasingly include both diseases, aiming to develop unified, mechanism-based treatment regimens that transcend traditional diagnostic boundaries [12,46].

Genetic and mRNA-based therapies are emerging as speculative but promising approaches for familial or monogenic forms of IgAN associated with IgA glycosylation defects. Advances in gene editing and mRNA technology may one day permit tailored correction of these abnormalities at their source, representing a new era of precision medicine in nephrology [34].

Finally, the growing importance of real-world data and longitudinal registries cannot be overstated. Initiatives such as CureGN and other national glomerular disease databases will be pivotal in establishing the long-term safety, efficacy, and cost-effectiveness of emerging therapies for IgAN. These platforms capture broader and more diverse patient populations than traditional clinical trials, thereby providing insights into treatment responses and adverse effects in routine clinical practice [46].

Overall, the future of IgAN management is moving decisively toward a personalized, precision-medicine framework. Patients will increasingly be stratified according to dominant pathogenic mechanisms, such as complement activation, mucosal immune dysregulation, or autoantibody-mediated disease, and receive targeted therapy combinations tailored to those profiles. Biomarkers, molecular diagnostics, and dynamic risk calculators will guide both the selection and timing of interventions. As clinical trials expand and real-world evidence accumulates, interprofessional collaboration, patient engagement, and equitable access to novel therapies will be crucial to translating these scientific advances into meaningful long-term outcomes for patients worldwide.

## Conclusions

IgAN has changed dramatically in the past decades, from a disease treated mostly by supportive care and non-specific immunosuppression to one with a growing list of targeted therapies. It is due to enhanced knowledge of disease pathogenesis, including galactose-deficient IgA1, mucosal immune dysregulation, and complement activation, that new, mechanism-based therapy has been developed. These agents include sparsentan, targeted-release budesonide, and iptacopan, which are the first FDA-approved medications solely for IgAN and have demonstrated outstanding efficacy in lowering proteinuria and maintaining kidney function. Concurrently, individualized treatment is still the priority. Immunosuppression and high-tech treatments are not necessary for all patients, and clinical judgment is balanced against the degree of disease, comorbidities, patient desire, and availability of treatments. Risk stratification models like histopathologic score and web-based prediction models are used to help decide, but cannot be relied on infallibly. Shared decision-making and vigilant monitoring are applicable, particularly due to the chronic nature of the disease and potential side effects of therapy.

Looking out to the horizon, the coming years will see the management of IgAN focusing on precision medicine, i.e., individualized treatment according to risk stratification, biomarkers, and molecular profiles. Ongoing clinical trials, real-world registries, and collaborative global research will keep bringing best practices forward. With ongoing innovation coupled with a patient-oriented approach, the future for patients with IgAN is brightening day by day.
